# Retraction: The regulation of HBP1, SIRT1, and SREBP-1c genes and the related microRNAs in non-alcoholic fatty liver rats: The association with the folic acid anti-steatosis

**DOI:** 10.1371/journal.pone.0345145

**Published:** 2026-03-18

**Authors:** The PLOS One Editors

After this article [1] was published, concerns were raised that the Fig 4 Control panel in this article [1] appears similar to Fig 8A in [2], despite [1] and [2] describing different control diet conditions. Author MAK stated that the same control animals were used for histological analysis in [1] and [2], the control diet used was the same in both studies, and the control diet reported in [1] is correct whilst the control diet conditions reported in [2] are incorrect.

During post-publication discussions, author MAK provided underlying image data, quantitative data, and the IACUC ethical approval document for the study in [1]. Upon editorial review of this information, additional concerns were identified. Specifically:

- The IACUC approval document appears to be dated after the animal studies started and there are concerns with the independence of the approval provided;
- The underlying replicate image data not published in [1] for the Fig 4 Control panel appear similar to Fig 8F in [2];
- Regarding the quantitative data provided underlying Tables 2 and 3, there are concerns with the calculations, statistical analysis, and alignment with the values in Tables 2 and 3 in [1].

Regarding the IACUC approval, author MAK stated the animal study was commenced whilst ethical approval was under review. As prior approval for the animal experiments was not received, the article does not comply with PLOS Animal Research policy.

Author MAK stated errors occurred in the data processing and preparation of Tables 2 and 3, and they provided updated versions of these tables. Upon editorial review, the *PLOS One* Editors identified additional concerns that not all results reported as statistically significant in Table 2 in [1] were reported as significant in the updated Table 2.

In light of the above concerns that question the reliability and integrity of the reported results, and compliance with the PLOS Animal Research policy, the *PLOS One* Editors retract this article.

MAK and HMRH did not agree with the retraction. MAK apologizes for the issues with the published article. HMRH stands by the article’s findings. MS, SEHEN, AHAI, SU, AAG, and IMEG either did not respond directly or could not be reached.

## References

[1] Salman M, Kamel MA, El-Nabi SEH, Ismail AHA, Ullah S, Al-Ghamdi A, et al. RETRACTED: The regulation of HBP1, SIRT1, and SREBP-1c genes and the related microRNAs in non-alcoholic fatty liver rats: The association with the folic acid anti-steatosis. PLoS One. 2022;17(4):e0265455. doi: 10.1371/journal.pone.0265455 35417465 PMC9007334

[2] Oriquat G, Masoud IM, Kamel MA, Aboudeya HM, Bakir MB, Shaker SA. The Anti-Obesity and Anti-Steatotic Effects of Chrysin in a Rat Model of Obesity Mediated through Modulating the Hepatic AMPK/mTOR/lipogenesis Pathways. *Molecules*. 2023;28(4):1734. doi: 10.3390/molecules28041734 36838721 PMC9962978

